# Calibration, Validation and Utility of ARTEMIS‐A: A Web Application for Early Identification of Mental Health Difficulties in Secondary Schools

**DOI:** 10.1002/mpr.70104

**Published:** 2026-08-20

**Authors:** Anne‐Marie Burn, Jan Stochl, Joanna Anderson, Aslihan Baser, Peter B. Jones, Tamsin Ford

**Affiliations:** ^1^ Department of Psychiatry University of Cambridge Cambridge UK

**Keywords:** assessment, computerised adaptive testing, mental health, schools, young people

## Abstract

**Background:**

Early identification can accelerate access to effective support. We aimed to calibrate and validate ARTEMIS‐A, a computerised adaptive assessment of mental health difficulties for young people (YP), and to explore its acceptability and feasibility.

**Method:**

The ARTEMIS‐A item bank comprises 175 questions from established standardised mental health measures. Calibration involved pupils aged 11–16 years completing overlapping sets of 40 questions. Convergent validity was evaluated by comparing calibrated ARTEMIS‐A scores with the Development and Well‐Being Assessment (DAWBA). Online interviews with YP, parents and school staff explored perceived benefits and harms.

**Results:**

Of 3183 recruited pupils from nine schools, 2276 provided complete calibration data. Then 103 YP from six schools completed both ARTEMIS‐A and the DAWBA. ARTEMIS‐A administered a median of four questions per participant (min 2, max 8), with a median completion time of 67 seconds. Convergent validity with clinically rated DSM 5 DAWBA diagnoses was good; correlation was 0.55 the area under the curve was 0.81. Parents and YP considered ARTEMIS‐A acceptable and feasible, highlighting its speed, simplicity, and device flexibility.

**Conclusions:**

ARTEMIS‐A is potentially an efficient, flexible and cost‐effective method of early identification of secondary school pupils who may require additional support for their mental health.

## 1 Background

An increasing number of young people (YP) experience mental health conditions (Ford 2020; Newlove‐Delgado et al. 2021; Sadler et al. 2018). Those affected struggle to cope with school and maintain positive relationships, while their outcomes in adulthood are frequently poor (Costello and Maughan 2015; Sellers et al. 2019; Thompson et al. 2023). Early identification of YP with mental health difficulties may accelerate their pathway to support and hence reduce the impact of mental health conditions on their current and future life trajectories (Anderson et al. 2019).

ARTEMIS‐A is a web platform (app) for accurately and rapidly assessing mental health difficulties in secondary school pupils. The ARTEMIS‐A prototype uses computerised adaptive testing technology (CAT) (Stochl et al. 2021), and was derived from a sample of adolescents and young adults (aged 14–28 years) (Stochl 2020; Wiedemann et al. 2023). The CAT algorithm was originally based on responses to an item bank comprising 106 questions from six standardised fixed‐length psychological measures. Completion time varied by age of respondent, with the prototype assessment typically completed in 1–7 minutes, far quicker than any of the underpinning six assessments (Stochl 2020). CAT enables instant automated scoring and tailored reports for pupils and school staff, further reducing the time and resources required in screening (Gardner et al. 2004). ARTEMIS‐A includes an online management dashboard to simplify the distribution of assessments within the school, data collection, reporting, monitoring and signposting. Throughout this paper, we use the term *mental health difficulties* to refer to symptoms measured by ARTEMIS‐A. We use *mental health condition* to refer to a diagnosed disorder or one inferred using the clinically rated DAWBA.

We applied a user‐centred approach to design the ARTEMIS‐A app, incorporating two iterative design feedback cycles (Still and Crane 2017). Initial co‐production work included consultations with 32 pupils, parents, school staff and mental health professionals to understand their needs and preferences for the user interface and experience (Burn et al. 2022). ARTEMIS‐A potentially provides schools with a highly efficient, flexible and cost‐effective method of early identification of pupils who may require additional support for their mental health, either through universal screening or individual assessments. It could also be used to monitor the impact of events (e.g., exams, lockdowns), interventions or policy changes at class, year or whole‐school level.

### 1.1 Current Study

We aimed to optimise ARTEMIS‐A for the secondary school population and to explore its feasibility for use in mental health assessment. Our objectives were to: (i) expand the item bank to incorporate questions covering a broader range of common adolescent mental health difficulties and explore the psychometric properties of the expanded item bank (Stochl et al. 2021), (ii) calibrate the expanded item bank for the secondary school population, (iii) validate ARTEMIS‐A in secondary school pupils aged 11–16 years against a multi‐informant standardised diagnostic tool, the Development And Well‐Being Assessment DAWBA; (Goodman et al. 2000); and (iv) qualitatively explore the acceptability and feasibility of using ARTEMIS‐A in schools.

## 2 Methods

### 2.1 Ethics Approval

The Cambridge Psychology Research Ethics Committee approved the study (reference PRE.2022.051).

### 2.2 Patient and Public Involvement

We collaborated with a young people's advisory group comprising YP aged 9–19 years from diverse backgrounds, and a teacher advisory group of three school staff members with roles in school mental health. These advisory groups guided various aspects of the study, including implementation in schools and the review of public‐facing documents.

### 2.3 Participant Recruitment

We approached key contacts in state‐funded mainstream and alternative provision secondary schools (e.g., school mental health leads, heads of wellbeing teams). In schools that agreed to take part, all pupils in years 7–13 (aged 11–18 years) were invited to participate in both the calibration and validation phases. For the calibration phase, pupils received a link (sent by a member of the school staff) to an online Young Person's Information Sheet that included researchers' details and an invitation to contact them with any questions. Parents of pupils under the age of 16 received an email from the school including a Parent Information Sheet and an online opt‐out form. For the validation phase, given the additional demands of DAWBA completion, opt‐in parental consent with assent from the YP was required for those under the age of 16, while YP aged 16 or above provided their own consent, but could invite their parents to complete the DAWBA.

We provided summary reports to participating schools comparing their pupils' collated ARTEMIS‐A scores with those of the rest of the sample. All participating schools were offered the opportunity to use ARTEMIS‐A in their school for one academic year following the study.

### 2.4 Procedures

#### 2.4.1 Expanding the Item Bank and Calibration of ARTEMIS‐A in Secondary School Pupils

##### 2.4.1.1 Item Bank Composition

The expanded ARTEMIS‐A item bank contained 175 items drawn from ten self‐report measures used in adolescent mental‐health and wellbeing research. These comprised the Mood and Feelings Questionnaire (MFQ; Costello and Angold 1988, 29 items retained), Revised Children's Manifest Anxiety Scale (RCMAS; Reynolds and Richmond 1978, 24 items retained), short Leyton Obsessional Inventory for Children and Adolescents (Bamber et al. 2002, 11 items), Behaviours Checklist developed for the ROOTS cohort (Goodyer et al. 2010, 8 items retained), Rosenberg Self‐Esteem Scale (RSES; Rosenberg 1965, 10 items), Schizotypal Personality Questionnaire (SPQ; Raine 1991, 10 items retained), Revised Child Anxiety and Depression Scale (RCADS; Chorpita et al. 2000, 47 items), Generalized Anxiety Disorder 7‐item scale (GAD‐7; Spitzer et al. 2006, 7 items), Patient Health Questionnaire‐9 (PHQ‐9; Kroenke et al. 2001, 9 items), and EPOCH Measure of Adolescent Well‐Being (Kern et al. 2016, 20 items).

##### 2.4.1.2 Calibration Design and Overlapping Forms

**FIGURE 1:**
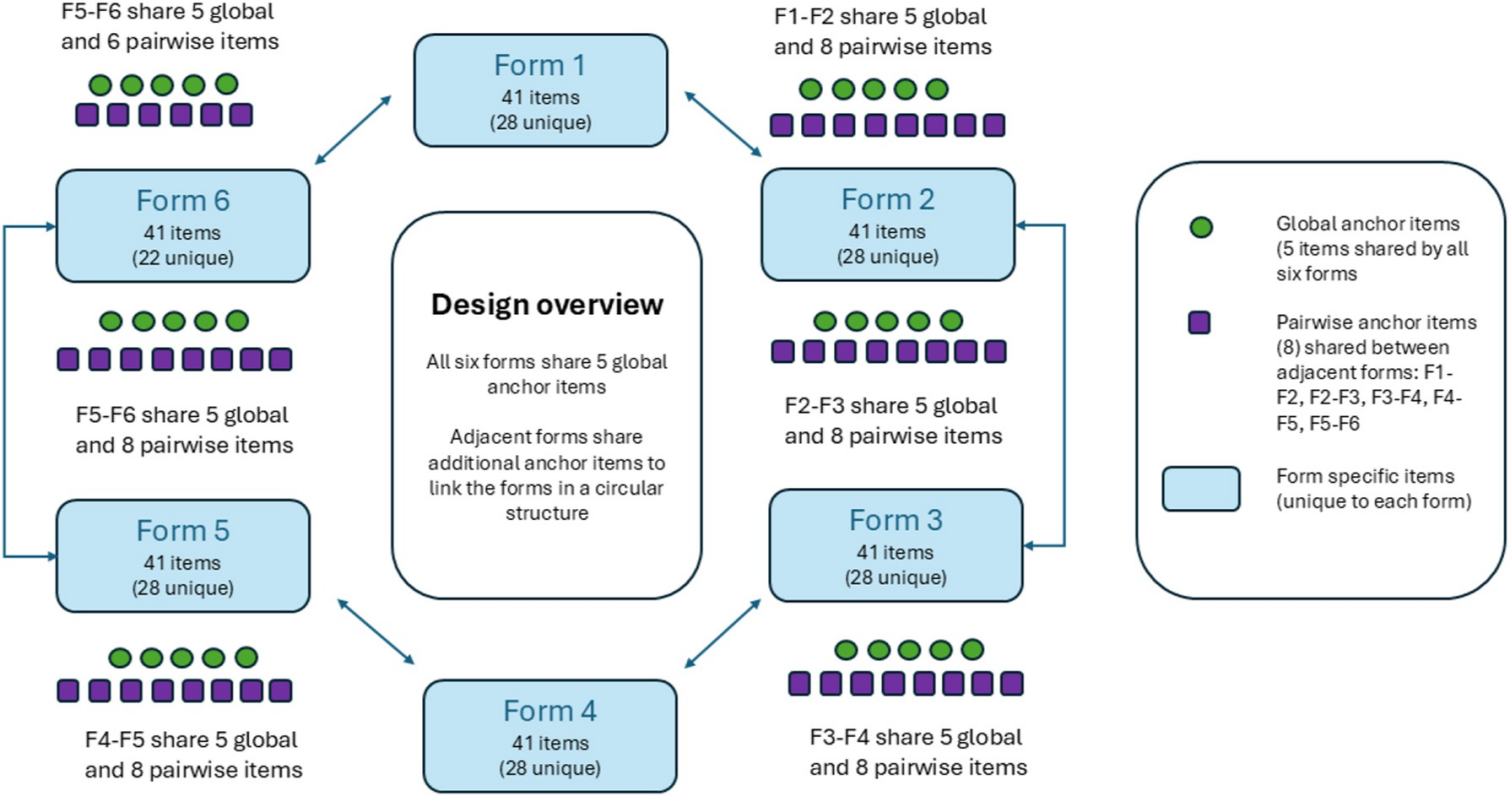
Connected common‐item multiple‐matrix sampling design used to calibrate the ARTEMIS‐A item bank. Five global anchor items were included in all six 41‐item forms. Adjacent forms were linked by eight additional pairwise anchor items, except Forms 1 and 6, which shared six additional items. Forms 1 and 6 contained 22 form‐specific items and Forms 2–5 contained 20 form‐specific items, producing a bank of 175 distinct items. Each pupil completed one form; items not assigned to that form were treated as planned missing data. Responses from all forms were analysed jointly using multidimensional item‐response theory modelling.

Administering all 175 items to every pupil would have imposed an unacceptable response burden. We therefore used a connected common‐item multiple‐matrix sampling design, illustrated in Figure 1. The item bank was distributed across six forms, each containing 41 items. Five global anchor items were included in every form. Additional pairwise anchor items connected adjacent forms in a circular structure: Forms 1–2, 2–3, 3–4, 4–5 and 5–6 shared eight additional items, while Forms 6 and 1 shared six additional items. Consequently, adjacent forms shared 13 items in total, except Forms 1 and 6, which shared 11 items, after inclusion of the five global anchors.

Forms 1 and 6 contained 22 form‐specific items, while Forms 2–5 contained 28 form‐specific items. Across the six forms, the design therefore covered 175 distinct items. Each participant completed one 41‐item form, representing a 76.6% reduction in the number of items administered relative to the complete item bank. Items not included in a participant's assigned form were treated as planned missing data rather than as omitted responses. The common‐item structure connected all six forms and enabled joint estimation of item parameters on a common latent scale.

Participants were randomly allocated to forms. We examined the distribution of school, age, sex and year group across forms to assess whether form allocation introduced systematic differences between the respondent groups.

##### 2.4.1.3 Psychometric Calibration

All observed item responses from the six forms were analysed jointly using exploratory multidimensional item‐response theory modelling. Unadministered items made no contribution to the individual participant's likelihood but contributed to estimation through responses from pupils assigned to the forms on which those items appeared. Ordered item responses were modelled using Samejima's graded response model (Samejima 1968).

Models containing one to seven total factors were estimated. Multidimensional solutions were subjected to bifactor rotation, allowing all items to load on a general factor representing psychological distress and subsets of items to load on orthogonal specific factors representing residual covariance not explained by the general factor. Model selection considered the Akaike information criterion, Bayesian information criterion and sample‐size‐adjusted Bayesian information criterion, together with the magnitude and pattern of factor loadings and the substantive interpretability of the specific factors. A four‐factor bifactor solution, comprising one general and three specific factors, was retained. The specific factors did not display sufficiently coherent substantive content and were therefore treated as nuisance dimensions. The general‐factor component was retained as the operational ARTEMIS‐A psychological‐distress metric, and the resulting item discrimination and threshold parameters were supplied to the computerised adaptive testing algorithm.

#### 2.4.2 Validation of the Improved ARTEMIS‐A Measure Against a Gold‐Standard Diagnostic Measure

We compared ARTEMIS‐A scores from pupils aged 11–16 recruited from nine schools with the DAWBA to evaluate its performance convergent validity, calculate the specificity, sensitivity and prediction parameters of ARTEMIS‐A scores, and to determine the optimal threshold for action. The DAWBA has parallel versions for parents and YP aged 11 years or over as multiple informants improve diagnostic accuracy in this population (Goodman et al. 2000). It combines highly structured questions relating to the diagnostic criteria in ICD‐10 (World Health Organization 2016) and DSM‐5 (American Psychiatric Association 2013) with semi‐structured probes about problem areas (Goodman et al. 2000). Trained clinical raters reviewed the qualitative and structured responses from all informants about individual YP to assign clinically rated diagnoses according to DSM‐5 (American Psychiatric Association 2013). The clinical rating allows YP who may not directly fit current diagnostic criteria but have clinically impairing difficulties to be assigned “not otherwise specified” diagnoses. Clinical raters may also spot misleading structured data when an informant's qualitative report indicates they have misunderstood structured questions, and to balance conflicting accounts from YP and parents as they would in the clinic.

Polyserial correlation was calculated to evaluate convergent validity between ARTEMIS‐A scores and DAWBA clinical ratings. To evaluate ARTEMIS‐A's performance against the standardised diagnostic tool we calculated Area Under Curve (AUC).

#### 2.4.3 Experience of Using ARTEMIS‐A and the DAWBA

We invited YP to take part in online individual or group interviews to explore the acceptability and feasibility of ARTEMIS‐A, including perceived benefits and harms. YP under the age of 16 were given the option of having their parents join the interview. We also invited school staff from schools that had dropped out of the study to understand why they withdrew. Separate topic guides were developed for the interviews with YP, parents, and school staff (see supplementary Files 1 and 2). Interviews lasted up to an hour. Participants received a £15 shopping voucher for their time and were provided with a list of mental health resources in a debrief email.

Interviews were recorded, transcribed, and thematically analysed using the Framework Method (Gale et al. 2013). Transcripts were checked for accuracy and anonymised before being analysed. An analysis plan broadly followed these stages: transcription; familiarisation with the interview; coding; development of a working analytical framework; application of the framework; charting of data into the framework matrix and interpretation of the data. Two members of the team (A‐MB, AB) began the analysis by familiarising themselves with the transcripts and using a combination of deductive and inductive coding. They met regularly to discuss how the codes could be grouped into categories, and these formed an initial working analytical framework. The working analytical framework was then applied to all transcripts. Once all the transcripts had been coded, Excel framework matrices were generated to chart the data by creating detailed summaries in each cell while retaining key verbatim quotes.

## 3 Results

### 3.1 Calibration

We recruited 3183 pupils aged 11–18 years from 9 schools, but calibration involved 2276 pupils with complete data on all presented questions. Investigation of the dimensionality of the improved item bank converged on a bifactor structure with 1‐general (interpreted as general mental difficulties) with high factor loadings and 2‐3 specific factors, confirming the essential unidimensionality and structural validity of the item bank for assessment of mental health difficulties. Specific factors had no clear interpretation and hence we considered them nuisance factors (factor loadings available on request). Fit indices of exploratory models are provided in Table 1. Item parameters of the 4‐factor bifactor model estimated via the Item Response Theory (Reise and Moore 2023) graded response model (Samejima 1968) were then used as input for the adaptive testing algorithm. The adaptive algorithm was then enabled and used for assessing mental health difficulties of YP during the validation stage.

**TABLE 1:** Fit indices of exploratory MIRT models with bifactor rotation.

Total number of factors^a^	AIC	SABIC	HQ	BIC
1	161,548	163,139	162,850	165,118
2	159,448	161,483	161,114	164,015
3	157,968	160,444	159,995	**163,526**
4	157,436	**160,352**	**159,823**	163,980
5	157,083	160,436	159,828	164,607
6	156,965	160,751	160,065	165,463
7	**156,882**	161,099	160,335	166,348

*Note:* The lowest values (in bold) indicate the best fitting model.

^a^ In bifactor model, 1 factor is always a general factor. Therefore, the number of specific factors in the model is total number of factors −1.

### 3.2 Validation

**FIGURE 2:**
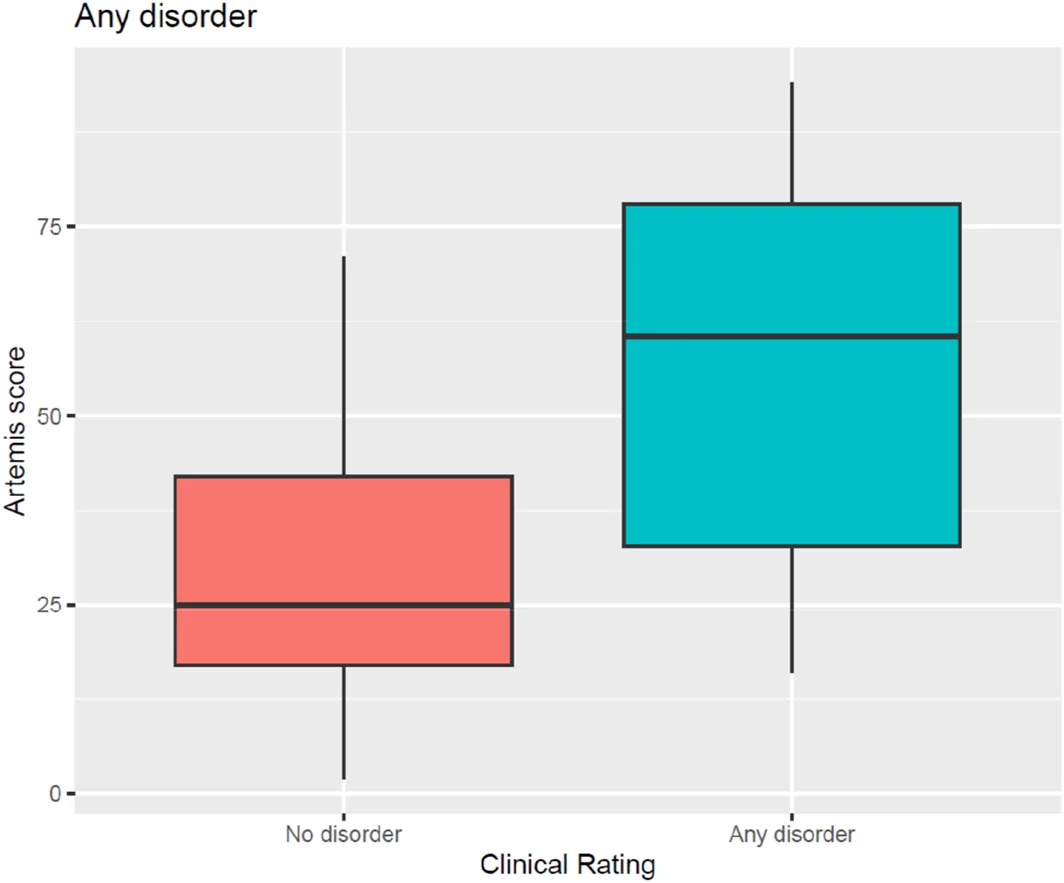
Distribution of ARTEMIS‐A scores across clinical ratings for any DSM 5 mental health disorders.

ARTEMIS‐A and DAWBA data for validation were collected for 103 YP from six schools. ARTEMIS‐A presented a median number of four items (min 2, max 8) while median completion time was 67 seconds. Raw scores approximated a normal distribution, as expected, and as illustrated by Figure 2 the distribution of ARTEMIS‐A scores differed by the presence or absence of disorder.

**FIGURE 3:**
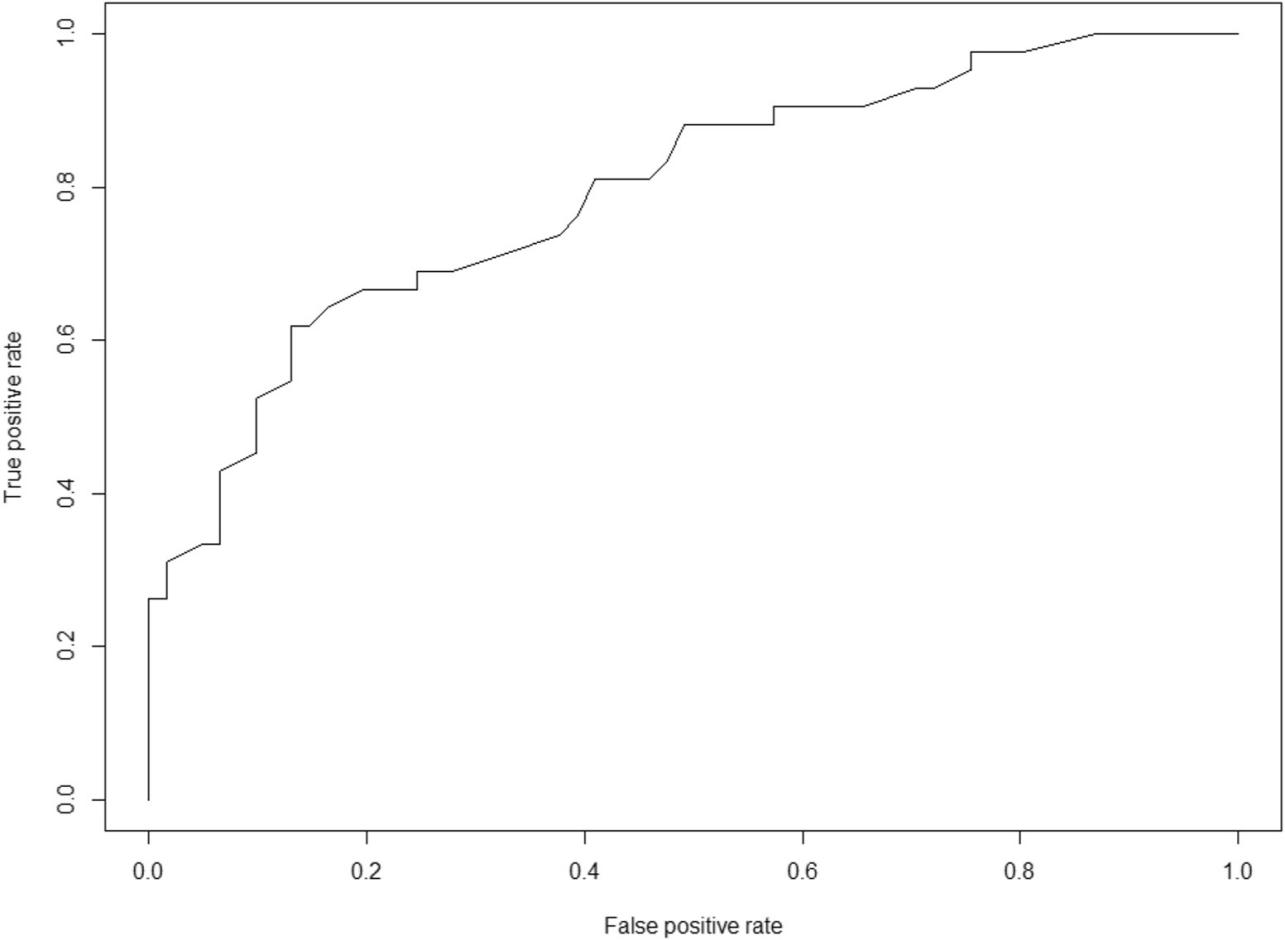
Performance of ARTEMIS‐A scores against DAWBA.

Convergent validity with clinically rated DAWBA was established by the comparison of ARTEMIS‐A scores against clinically rated DAWBA diagnosis of any mental health condition as outlined in Figure 3.

Figure 3 presents the receiver operating characteristic (ROC) curve illustrating the discriminative performance of ARTEMIS‐A against DAWBA as the reference standard. The ROC curve plots the true‐positive rate (sensitivity) against the false‐positive rate (1‐specificity) across all possible thresholds. The area under the curve (AUC) was 0.81, indicating good overall diagnostic accuracy. This value means that ARTEMIS‐A correctly ranks a randomly selected child with clinical mental health condition higher than a randomly selected YP without a mental health condition in approximately 81% of cases. The curve rises steeply towards the upper‐left corner, suggesting high sensitivity with relatively low false‐positive rates across most thresholds.

These results demonstrate that ARTEMIS‐A provides reliable discrimination relative to structured diagnostic assessment, supporting its potential utility as a rapid, scalable screening and triage tool for children's mental health services.

### 3.3 Qualitative Interviews

**FIGURE 4:**
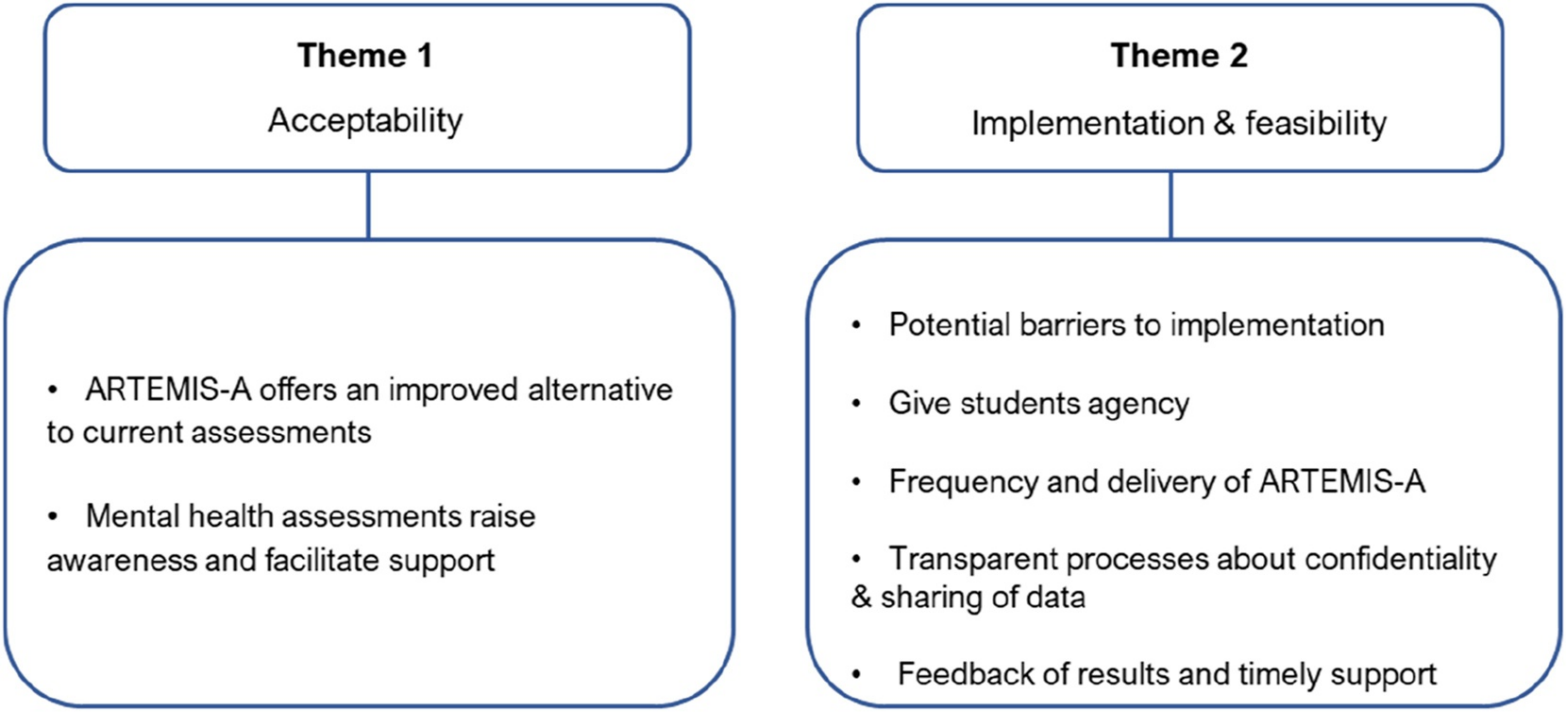
Themes and subthemes.

We recruited 15 participants. The sample included 10 YP (5 females, 5 males) aged 11–15 years old (median age = 15), recruited from four participating schools. All YP had completed both ARTEMIS‐A and the DAWBA. Additionally, we recruited three parents, each from a different participating school. We interviewed two staff members from different schools that had withdrawn from the study to understand their decisions. Administrative delays meant that recruitment and interviews took place during the summer break, which made recruitment challenging. The analysis identified two overarching themes which are described below with illustrative quotes from YP, parents and school staff (Figure 4).

#### 3.3.1 Theme 1: Acceptability

##### 3.3.1.1 ARTEMIS‐A Offers an Improved Alternative to Current Assessments

Participants liked ARTEMIS‐A's simple design, quick and dynamic interface, and the flexibility to complete the assessment on various devices at their convenience. YP said they found ARTEMIS‐A quick and easy to use and considered it an improvement on existing approaches to school mental health assessment.

…they have wellbeing quizzes sent out every few weeks, but they're not very like clear. And the questions are quite like related to one topic. I think ARTEMIS would be quite good because it's a variation of different things.

(YP)

The adaptive nature of the assessment, whereby questions changed depending on previous answers, was highlighted as an important feature that could help maintain students' engagement with repeated assessments.

I think key for me is, it's got to be simple. It's got to be straightforward. I think it's got to be quick for the kids to fill in. And if the questionnaire changes, which you say it does, I think that's key, because that gets people engaged with it.

(Parent)

YP reported completing the assessment in a shorter time than they had expected; some said the brevity of the assessment would benefit those who found it difficult to sustain attention.

Think it was good that it was quite brief…because if you have like a short attention span, then it's good for that.

(YP)

##### 3.3.1.2 Mental Health Assessments Raise Awareness and Facilitate Support

Both parents and YP said it was reassuring that their schools were proactively monitoring students' mental health, noting that assessments would raise awareness and encourage students to reflect on their well‐being. They believed that regular school‐based mental health assessments would serve as a confidential check‐in mechanism, helping to identify students in need of support, especially since mental health can fluctuate throughout the academic year.

It would be important, because I've just done my final exams. I was perfectly fine when I was doing my mocks or whatever. But then that skyrocketed my anxiety when my finals came up. Yeah, I guess it would be useful to monitor more how people change based on different types of stress.

(YP)

Additionally, regular check‐ins would provide school wellbeing teams with valuable data on student mental health and help prompt necessary support.

I think the whole idea of it is brilliant and it definitely helps schools to have something like this available because there is so much need at the moment…. we would be able to, quite quickly, get an understanding of where our students were in terms of their mental health and wellbeing.

(Staff)

I think it would make like teachers and pastoral managers more aware of people's situations. I've seen multiple people get really anxious about things, but there's no way of them really reaching out teachers, because they're worried that they're not going to be taking taken seriously.

(YP)

#### 3.3.2 Theme 2: Implementation and Feasibility

##### 3.3.2.1 Potential Barriers to Implementation

School staff thought ARTEMIS‐A was a useful assessment tool but highlighted several concerns. Staff faced some technological challenges in distributing the assessment, including issues with platform access and expired links. Additionally, staff raised the issue that some parents may not provide consent for their child to complete the assessment or that it might raise parents' expectations about the level of support that schools could realistically provide. Staff voiced concerns about ARTEMIS‐A increasing the number of YP identified as having mental health needs, and schools being unable to provide adequate support due to limited capacity and resources.

One staff member explained that their school had withdrawn from the study over concerns about assessment questions relating to self‐harm and suicidal thoughts.

So I went in to a year nine form group that were trialling it within that form group, there was quite a number of students who had self‐harmed, had actually tried to take their own life, had other needs like special educational needs. And some of the questions that were coming up in the calibration tool, I was just quite concerned about.

(staff)

##### 3.3.2.2 Give Students Agency About Where and When to Complete

To ensure successful implementation, parents and YP emphasised that students should be able to decide where and when to complete ARTEMIS‐A. One parent noted that some students may feel obligated to complete the assessment if it is sent via school email. The consensus was that students should be able to decline and should not feel pressured to complete ARTEMIS‐A if they feel uncomfortable about doing so.

YP expressed concerns about privacy if completing the assessment at school, as they might feel embarrassed or worry that others could see their responses. Both staff and YP commented that some students might not take the assessment seriously.

…at my school you're not allowed phones, so we'd only be doing it on a laptop or computer and then that's quite big screens. Anybody could look and easily see what they're doing.

(YP)

One parent suggested that providing a private space at school could be beneficial, especially for students who may not feel safe or supported at home. They highlighted the importance of schools managing this sensitively, particularly if the assessment was targeted, to avoid stigmatising those who are asked to complete it.

Overall, YP said they would feel more comfortable completing the assessment at home, where they would have greater privacy and a lower risk of being teased by peers, allowing them to respond more honestly.

My only concern was like will people answer honestly if they did it in school versus if they did at home.

(YP)

##### 3.3.2.3 Frequency and Delivery of ARTEMIS‐A

Given its speed and ease of use, YP said they would be willing to complete ARTEMIS‐A regularly, suggesting that termly or monthly assessments would be ideal.

I think our school will do it once a term and I think that's quite good… once or twice every term will be quite good.

(YP)

Most participants were comfortable with school‐wide screening, suggesting it would normalise the process and reduce any embarrassment associated with seeking help. Some expressed concerns about potential stigma or the risk of missing valuable information if the assessment were limited to certain individuals or groups. One suggestion was for schools to adopt a phased approach, starting with universal distribution of the assessment and then adjusting the frequency based on the results and key times in the academic year.

I think it should be given to all the kids because some kids aren't always visibly showing something or they might not notify the school about it. And they might be trying to hide it from people and Artemis might be a good way for them to tell the school without it being too much of like a big thing.

(YP)

##### 3.3.2.4 Transparent Processes About Confidentiality and Sharing of Data

Both YP and parents emphasised the importance of keeping the data captured through ARTEMIS‐A private and confidential. YP felt strongly that their data should be deleted when they leave their school, highlighting the need for schools to establish a clear and transparent process for data governance, ensuring that students are informed about who operates the ARTEMIS‐A system, who has access to the information and how it is used.

But as long as they're not, I guess using it for something other than trying to support students.

(YP)

Once you've left school, I think if they kept it afterwards, there's no need for them to keep it [and] that would be maybe worrying and probably illegal.

(YP)

YP expressed concerns about stigma or judgement if their assessment results were shared with other staff and they recommended that access to data be limited to staff directly involved in student support. Generally, they felt comfortable with results being accessible to school staff in pastoral or wellbeing roles or staff members who they considered ‘trustworthy’.

Whoever they trust really—if they don't trust someone, why would they want them to know? Yeah, it will be useful to know who's got what [access].

(YP)

##### 3.3.2.5 Feedback of Results and Timely Support

YP believed that receiving an assessment result would help them understand their mental health status. They would prefer prompt feedback, ideally on the same day or the following day to prevent self‐diagnosis and unnecessary anxiety. Both YP and parents discussed the importance of timely feedback for students who receive a ‘red flag’ result, and emphasised the need for clear communication and robust support systems. Parents said they would only want to be notified if their child's score indicated that they were at risk. They stressed the importance of ensuring students felt comfortable and involved throughout the assessment process, while also providing necessary support and information to parents.

…the scoring system is quite good and it's quite easy to understand the scores and put into perspective anyway. So, they [schools] might see who's struggling, who's not, who's okay, and it's quite simple for schools to understand.

(YP)

Participants discussed the importance of seeking the YP's consent before sharing their assessment results with parents and emphasised the need for ongoing communication between schools, students and parents regarding support.

I would like to know if my child is feeling distress, if my child is battling some mental challenges. So, I definitely would like to be informed… it would help to have that ongoing cooperation with the school if the help is needed for child, I think it should come from both parents and school. We need to be involved. And I would also like to know if there are any potential resources I could use, and people I could go to, to help my child. But at the same time, I would like my child to be involved in these conversations as well. So I wouldn't do anything behind her back.

(Parent)

## 4 Discussion

We recruited sufficient schools and pupils to allow recalibration of the expanded ARTEMIS‐A item databank and have produced an algorithm that is quicker to resolve and requires even fewer questions from participants. The agreement with the clinically rated diagnoses of any disorder was satisfactory, especially given that in a school setting aiming at early identification and intervention, the presence of a possible disorder (pre‐diagnosis) may be of more relevance than identifying only those with a probable diagnosis. We are currently evaluating sensitivity to change over time in a sample attending Mental Health Support Teams in schools and Children and Young People's Mental Health Services. Consistent with previous research, initial feedback from those interviewed about ARTEMIS‐A was positive regarding its feasibility, acceptability and utility (Burn et al. 2022; Lan et al. 2025). However, careful consideration is required in how ARTEMIS‐A is implemented, particularly in ensuring the appropriate completion process and controlling who has access to reports and data. Similar to other studies, a key concern raised is the importance of safeguarding the privacy and confidentiality of students' mental health data (Carey et al. 2024).

ARTEMIS‐A is the first mental health assessment to be made available for YP using CAT in the UK (Stochl et al. 2021). A strength of the app is that it was co‐produced with YP, parents and school staff to ensure it is user‐friendly and easy to use (Burn et al. 2022). Convergent validity against the gold‐standard multi‐informant standardised diagnostic tool was established but investigation of individual mental health conditions in a larger sample is required. The expanded item bank now covers a broader range of mental health difficulties that are common among secondary school pupils, and measures that are more appropriate to this age group.

There are some methodological limitations. We recruited a smaller sample than initially planned at both the calibration and validation stages, but these smaller datasets were sufficient to recalibrate the algorithm, and we have clearly demonstrated the validity of ARTEMIS‐A against an established diagnostic measure. We had aimed to recruit more participants for interviews but, due to the school summer break recruitment was challenging. While the sample was smaller than expected, the interviews were in‐depth and provided rich data for analysis. Some schools withdrew from the study over concerns about assessment questions which ask about suicidal ideation and self‐harm, despite the non‐specificity of these questions, high frequency of these thoughts and feelings in this age group and the extensive use of these questionnaires with both clinical and population samples. Importantly, evidence shows that asking about suicidal ideation or self‐harm does not increase risk of harmful outcomes, and asking may provide relief or encourage help seeking (Polihronis et al. 2022).

ARTEMIS‐A provides schools with a brief, easy‐to‐use and practical tool that can accurately identify mental health difficulties in secondary school pupils. We will continue to work with participating schools who are using the newly calibrated tool and will seek feedback on their experience to inform guidance for schools implementing ARTEMIS‐A and further evaluation. Recent work has shown that ARTEMIS‐A could be a useful tool for health professionals working with YP in other settings such as primary care, community paediatrics and Children and Young People's Mental Health Services (Lan et al. 2025). Further work is required to demonstrate its sensitivity to change and whether there are distinct patterns in ARTEMIS‐A during the school year, as well as variations in the prediction of different types of mental health condition and suitable thresholds for concern and action.

## Author Contributions

**Anne‐Marie Burn:** conceptualization, funding acquisition, writing – review and editing, methodology, supervision, formal analysis, writing – original draft, investigation. **Jan Stochl:** conceptualization, funding acquisition, writing – review and editing, methodology, supervision, formal analysis, investigation. **Joanna Anderson:** conceptualization, funding acquisition, writing – review and editing, methodology, supervision, investigation. **Aslihan Baser:** writing – review and editing, formal analysis, investigation. **Peter B. Jones:** conceptualization, funding acquisition, writing – review and editing, methodology, supervision, investigation. **Tamsin Ford:** conceptualization, funding acquisition, writing – review and editing, methodology, supervision, writing – original draft, investigation, formal analysis.

## Funding

This work is funded by NIHR Invention for Innovation i4i Connect 5 & Children and YP's Mental Health Business Plan (NIHR203834).

## Conflicts of Interest

J.S. and P.B.J. are co‐founders of Cambridge Adaptive Testing Ltd. Tamsin Ford's research group receives research funding for research method consultation from Place2Be, a third sector organisation providing mental health training and intervention in schools. Neither Place2Be nor the schools they work with were involved in this study.

## Supporting information


Supporting Information S1

Supporting Information S2

## Data Availability

The data that support the findings of this study are available on request from the corresponding author. The data are not publicly available due to privacy or ethical restrictions.
