# Supplementary material for: Calibration, Validation and Utility of ARTEMIS‐A: A Web Application for Early Identification of Mental Health Difficulties in Secondary Schools
Source: Int J Methods Psychiatr Res. 2026 Aug 20;35(3):e70104. doi: 10.1002/mpr.70104 (PMC13491535; doi:10.1002/mpr.70104)
Supplement: Supplementary file 1 [file MPR-35-e70104-s001.pdf]

Supplementary File 1: Topic Guide for Interviews/Focus Groups with Young People

ARTEMIS-A Focus group/interview: Young people

Purpose: To explore students' views about benefits and concerns with the use of ARTEMIS-A, including how they think it should be used.

Aim: to conduct 2 focus groups with YP (5-7 in each group) up to 60 minutes. Or 8-10 interviews about 30-40 minutes

Mode: Either face-to-face or online

Equipment

- Participant Information Sheet
- Consent Form
- Demographic Form

Pre-Focus Group/Interview

- Participants have read and understood the Information Sheet
- Researcher has obtained informed consent from young people aged 16+. For those aged 14-16 years old, assent has been obtained, as well as parental consent.

Introduction:

- Research team will begin by introducing themselves and thank participants for agreeing to take part in the study.
- Explain how the focus group/interview will run, we will be able to send a voucher as a thank you.
- It will be recorded - explain confidentiality.
- For online groups, ask Ps to be in a quiet private space and that we'd prefer cameras stay on.

Introductions from facilitators and go around the participants. Short recap of the Artemis project

<table>
<tr><td>Key area</td><td>Interview question</td></tr>
<tr><td>Acceptability</td><td>1. You used the Artemis app and it would be helpful to know what you think about it. This can include good and bad things, things about the web-app itself or how it could be used in your school (can show Artemis on slide and then table).

<table>
<tr><td>Things I didn’t like about Artemis</td><td>Things I’m not sure about</td><td>Things I liked about Artemis</td></tr>
<tr><td>[insert comments using shared screen or whiteboard]</td><td></td><td></td></tr>
</table>

Prompts:

Reassurance about mental health (peace of mind)
More likely to speak about mental health at school (conversations about MH will become normal)
Students can let school staff know how they are feeling (it’s a communication tool)
It’s quick and easy to use
Can be given to everyone and track how they are doing over time
</td></tr>
<tr><td>Implementation</td><td>2. Did you use Artemis on a desktop computer or on your mobile? Why?</td></tr>
<tr><td></td><td>3. Where did you complete it? Home, school, elsewhere? (if FG, note numbers)
Probe: issues around convenience, comfort, privacy</td></tr>
<tr><td>Experience</td><td>4. How did you feel after completing the assessment?</td></tr>
</table>

	Probe: whether it was reassuring or created anxiety
	5. What information would you like to know from your school after the assessment?
	6. What did you think of the feedback report? May not have had this….<br>*Was it easy or difficult to understand? How could it be improved?*
	7. How do you feel about school staff receiving your assessment results? If this were used by schools who should be able to see the results e.g. form tutors, well being team, any member, school nurses<br>*Probe: concerns about confidentiality of data; or benefits of communicating feelings of distress to staff, are there specific staff you like to receive information about results of your assessment?*<br>How would you like information about your assessment to be given back to you?<br>*Feedback from a member of school staff about the assessment results? If yes, which member of the school staff would you like to discuss the results of your assessment if needed?*
	8. Do you think school staff should inform parents? *Probe: for any concerns*
	9. You completed two questionnaires for the project and it would be helpful to hear what you think about them.<br>Let's start with the DAWBA (if needed, show slide to remind them). How did you find completing this questionnaire?<br>Probe: what did they like, didn't like

Check with participants whether there is anything else that they would like to say, which hasn't already come up in the focus group. Thank participants for taking part. Issue vouchers and offer an opportunity to ask any further questions participants may have.
