# Supplementary material for: Calibration, Validation and Utility of ARTEMIS‐A: A Web Application for Early Identification of Mental Health Difficulties in Secondary Schools
Source: Int J Methods Psychiatr Res. 2026 Aug 20;35(3):e70104. doi: 10.1002/mpr.70104 (PMC13491535; doi:10.1002/mpr.70104)
Supplement: Supplementary file 2 [file MPR-35-e70104-s002.pdf]

Supplementary File 2: Topic Guide for Interviews/ Focus Groups with School Staff

ARTEMIS-A Focus groups/interviews: school staff

Purpose: To explore school staff's views about the potential benefits and concerns staff may have with the use of ARTEMIS-A. To understand how the app could be used in their school and any barriers to implementation.

Aim: to conduct 2 focus groups with staff (5-7 in each group) up to 60 minutes. Or, 8-10 interviews about 30-40 minutes

Participants: 5-7 school pastoral staff (including school counsellors) will be recruited from participating schools.

Mode: Either face-to-face or online

Equipment

- Participant Information Sheet
- Consent Form
- Demographic Form

Pre-Focus Group/Interview

- Participants have read and understood the Information Sheet
- Researcher has obtained informed consent

Notes:

- Research team will begin by introducing themselves and thank participants for agreeing to take part in the study.
- Explain how the focus group/interview will run, we will be able to send a voucher as a thank you.
- It will be recorded - explain confidentiality. Remind participants they are able to stop / pause / choose not to answer any questions
- We will have some screenshots of Artemis to show either on screen or in print outs. *Prompts* are provided but may not be needed.

Introductions from facilitators and go around participants. Short recap of the Artemis project - explain that we would like to know what they think of the app and how it might be used in their schools

**Key area**	**Interview question**
Benefits	1. We'd like to know what you think about the Artemis app. What do you think the benefits would be for schools using it?<br>• *Speed, easy to use*<br>• *track how pupils are doing over time*<br>• *Can identify those who are 'under the radar'/not picked up*<br>• *MH becomes part of school culture*
Concerns	2. Do you have any concerns about using Artemis to assess student mental health in your school?<br>• *type of question i.e. sensitive topic, might make YP feel more anxious*<br>• *Worried about stigma (YP being singled out)*<br>• *Lack of privacy at school*<br>• *Students not completing properly*<br>• *Too many pupils get identified and not enough help/support*<br>• *Worried about confidentiality of their data and who will access it*<br>• *Telling parents about their results/involving parents*
Implementation	*3.* How do you think your school would use Artemis?<br>• *Universal vs selective approaches*<br>• *Will school expect students to complete at school or home*<br>• *How will Artemis fit with your existing mental health support practices?*

	4. Just a reminder of the automated report that schools receive (show slide/printout as a reminder)<br><br>• *Is this useful?*<br>• *Any suggestions to change/improve it?*
	5. How should we give data back to individuals?
	6. What do schools want the YP to know?
	7. How do you think Artemis will fit with the values and working practices of the school?<br><br>• *SLT on board?*<br>• *Do you anticipate any barriers?*

Check with participants whether there is anything else that they would like to say, which hasn't already come up in the focus group.

Thank participants for taking part. Issue vouchers and offer an opportunity to ask any further questions participants may have.
